# Immunogenicity and pre-clinical efficacy of an OMV-based SARS-CoV-2 vaccine

**DOI:** 10.21203/rs.3.rs-2788726/v1

**Published:** 2023-05-25

**Authors:** Alberto Grandi, Michele Tomasi, Irfan Ullah, Cinzia Bertelli, Teresa Vanzo, Silvia Accordini, Assunta Gagliardi, Ilaria Zanella, Mattia Benedet, Riccardo Corbellari, Gabriele Di Lascio, Silvia Tamburini, Elena Caproni, Lorenzo Croia, Micol Ravà, Valeria Fumagalli, Pietro Di Lucia, Davide Marotta, Eleonora Sala, Matteo Iannacone, Priti Kumar, Walther Mothes, Pradeep D. Uchil, Peter Cherepanov, Martino Bolognesi, Massimo Pizzato, Guido Grandi

**Affiliations:** 1: Toscana Life Sciences Foundation, Via Fiorentina 1, 53100, Siena, Italy; 2: BiOMViS Srl, Via Fiorentina 1, 53100, Siena Italy; 3: University of Trento, CIBIO Department, Via Sommarive 9, 28123, Trento Italy; 4: Department of Internal Medicine, Section of Infectious Diseases, Yale University School of Medicine, New Haven, CT 06520, USA; 5: IRCCS San Raffaele Scientific Institute, Division of Immunology, Transplantation and Infectious Diseases, 20132 Milan, Italy.; 6: Vita-Salute San Raffaele University, 20132 Milan, Italy.; 7: IRCCS San Raffaele Scientific Institute, Experimental Imaging Center, 20132 Milan, Italy.; 8: Department of Microbial Pathogenesis, Yale University School of Medicine, New Haven, CT 06510, USA; 9: The Francis Crick Institute, Chromatin Structure and Mobile DNA Laboratory, London, UK; 10: University of Milan, Via Celoria 26, 20122, Milan, Italy

## Abstract

The vaccination campaign against SARS-CoV-2 relies on the world-wide availability of effective vaccines, with a potential need of 20 billion vaccine doses to fully vaccinate the world population. To reach this goal, the manufacturing and logistic processes should be affordable to all countries, irrespectively of economical and climatic conditions.

Outer membrane vesicles (OMV) are bacterial-derived vesicles that can be engineered to incorporate heterologous antigens. Given the inherent adjuvanticity, such modified OMV can be used as vaccine to induce potent immune responses against the associated protein. Here we show that OMVs engineered to incorporate peptides derived from the receptor binding motif (RBM) of the spike protein from SARS-CoV-2 elicit an effective immune response in vaccinated mice, resulting in the production of neutralizing antibodies (nAbs). The immunity induced by the vaccine is sufficient to protect the animals from intranasal challenge with SARS-CoV-2, preventing both virus replication in the lungs and the pathology associated with virus infection. Furthermore, we show that OMVs can be effectively decorated with the RBM of the Omicron BA.1 variant and that such engineered OMVs induced nAbs against Omicron BA.1 and BA.5, as judged by pseudovirus infectivity assay. Importantly, we show that the RBM_438-509_ ancestral-OMVs elicited antibodies which efficiently neutralized *in vitro* both the homologous ancestral strain, the Omicron BA.1 and BA.5 variants, suggesting its potential use as a pan SARS-CoV-2 vaccine.

Altogether, given the convenience associated with ease of engineering, production and distribution, our results demonstrate that OMV-based SARS-CoV-2 vaccines can be a crucial addition to the vaccines currently available.

## Introduction

The dramatic SARS-CoV-2 pandemic exploded worldwide at the beginning of 2020 has triggered an unprecedented race to the development of effective vaccines. In less than a three-year timeframe, hundreds of vaccines have been designed and tested in the preclinical settings, more than 100 have reach the clinic, and some 24 are currently authorized for human use ^1^. It is estimated that more than 9 billion doses have been administered so far worldwide, saving approximately 1 million lives.

Despite this spectacular success of modern vaccinology, to paraphrase what Messala said to Giuda Ben-Hur on his deathbed, “the race is not over” (from the movie “Ben-Hur”, Director W. Wyler, 1959). Because of costs and logistic issues, vaccine distribution is heavily unbalanced, with half of the planet still waiting for a dose and with only 4% of populations in low-income countries being vaccinated ^2^. Moreover, SARS-CoV-2 has the extraordinary capacity to continuously accumulate mutations, which allow the virus to escape, at least partially, the host immune responses and, at the same time, to preserve its infectivity and virulence ^3^.

To overcome such challenges and to provide a sustainable long-term prophylaxis, a “panvaccine” capable of eliciting a broad, cross-protective immune response should become available. This would avoid the need of booster immunizations using vaccines tailored for the emerging variant-of-concern (VOC). In addition, the vaccine should rely on a production process easily scalable at low costs and should not require the cold chain, a situation which could otherwise make the vaccine logistically and economically prohibitive for several countries.

Among the several technologies available for vaccine development, outer membrane vesicles (OMVs) have emerged in recent years as an attractive tool capable of coupling excellent built-in adjuvanticity provided by the microbe-associated-molecular patterns (PAMPs) embedded in the vesicles, and an easily scalable production and purification process. Anti-Neisseria OMV-based vaccines are currently available for human use ^4^, and others against Shigella and Salmonella are in advanced clinical phases^5,6^.

We have recently developed a platform based on “proteome minimized” *E. coli* OMVs selectively loaded with heterologous antigens^7^. The platform has been successfully applied to design prophylactic vaccines against infectious diseases ^8^ and has been shown to stimulate potent anti-tumor activity in different mouse models ^9,10^.

Given that OMVs are readily phagocytosed, antigens carried by the vesicles are efficiently presented by professional antigen presenting cells, leading to the elicitation of both antibodies and T-cell responses, coupled to production of IFN-γ, ensuring a sustained Th1 response as well as an optimal humoral response. Since clinical evidence demonstrates that an accelerated induction of a Th1 cell response associates with less severe cases of COVID-19 ^11,12^ and that convalescent individuals develop strong memory CD4+ and CD8+ T cells ^13^, the ability of OMVs to trigger Th1 represents a desired feature. Crucially, in addition to the simple and cost-effective setup required to produce and purify OMVs^14^, the antigen-decorated vesicles are extremely stable for long-term storage at room temperature, making it a convenient vaccine to distribute all over the world.

Essentially all available vaccines and those under development are designed to induce antibodies specific for the spike (S) protein or its receptor binding domain (RBD). Neutralizing titres found in vaccinees, as well as in convalescent patients, correlate strongly with antibody binding to RBD, which binds the angiotensin-converting enzyme 2 (ACE2) on the host cell membrane^15^. Accordingly, the most potent monoclonal antibodies isolated from convalescent patients recognize epitopes located in RBM, the RBD interface with ACE2 (see Hwang et al.^16^ for a review). The ability to specifically concentrate the immune response against these epitopes would therefore exclusively elicit neutralizing antibodies, while minimizing generation of non-neutralizing or poorly neutralizing immunoglobulins binding to irrelevant spike regions.

The OMVs offer the unique opportunity to display short and defined epitopes to B- and T-cells in a highly immunogenic context provided by the bacterial vesicle. Taking advantage of such potential and the availability of the crystal structure of the RBD in complex with ACE2, we engineered the OMVs with peptides from the SARS-CoV-2 ancestral RBM. Here we show that RBM-derived peptides can be expressed in the OMV membrane and induce neutralizing antibodies. In particular, the RBM_438-509_ region elicits antibody titres sufficient to fully protect hACE2 transgenic mice from the challenge with SARS-CoV-2. Moreover, we show that the same region from the BA.1 variant can also be efficiently expressed in the OMVs, highlighting the plasticity of the OMV platform, which can be rapidly adapted to the emerging variants. Finally, and importantly, we show that the RBM_438-509_ ancestral-OMVs elicit antibodies which can neutralize *in vitro* both the homologous ancestral strain and the Omicron BA.1 variant. Since natural infection and vaccines based on the ancestral Spike protein induce antibodies with poor neutralization capacity to most VOCs, and particularly to the Omicron variant(s), the data suggest that in the context of OMVs the RBM region elicits a novel population of antibodies which have a broad, cross-reactive neutralization capacity.

Given the efficacy of the vaccine in the animal model, the cross-neutralization capacity, the ease of its engineering, the cost-effective production process, and the stability at room temperature, we propose the RBM_438-509_ ancestral-OMVs as a promising candidate to continue the vaccination campaign against SARS-CoV-2.

## Results

### Design and construction of the OMV-based vaccine

**Figure 1.**
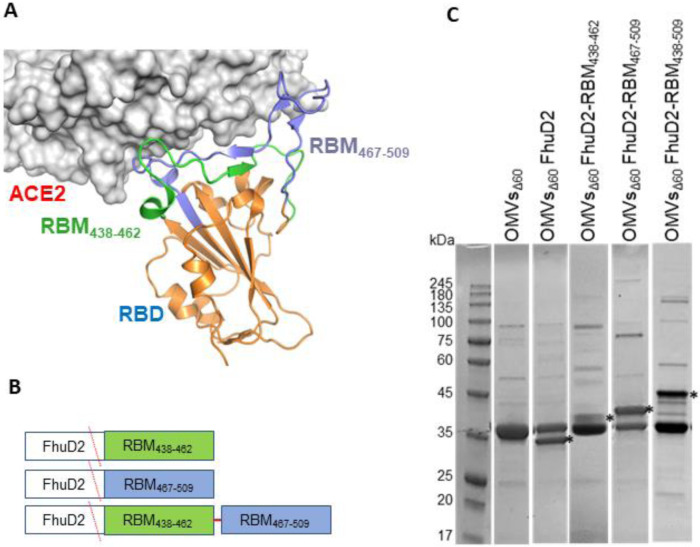
Construction and production of OMVs carrying SARS-CoV-2 RBM antigens (A) Topology of the interaction between SARS-CoV-2 RBD and ACE2 with the indication of the two RBM polypeptides tested in this study. (B) Schematic representation of the pET21-based constructs expressed in BL21(DE3)*Δ60* to decorate OMVs. *Staphylococcus aureus* Ferric hydroxamate receptor 2 (FhuD2) was fused to one copy of RBM_438-462_, RBM_467-509_ and RBM_438-509_ SARS-CoV-2 epitopes. (C) SDS-PAGE of purified OMVs derived from BL21(DE3)*Δ60* cultures expressing pET21-FhuD2-RBM_438-462_, pET21-FhuD2-RBM_467-509_ and pET21-FhuD2-RBM_438-509_ plasmids or the control pET21-FhuD2 or empty pET21 plasmids. Stars indicate the predicted migration of the fusion proteins.

As shown by the 3D structure of the SARS-CoV-2 RBD in complex with ACE2, the concave receptor binding motif (RBM) of the spike protein is organized in two discrete ordered chains incorporating the β5 and β6 strands which cross each other and include most of the residues contacting ACE2 (Figure 1A). Accordingly, patient-derived monoclonal antibodies (mAbs) binding these RBD chains potently neutralize virus cell entry *in vitro* and protect different animals from viral challenge ^17-21^, confirming the crucial role of RBM in engaging the receptor and demonstrating its immunogenicity *in vivo.* These mAbs are currently in clinical use or in advanced clinical development^22^.

In the attempt of producing a vaccine eliciting neutralizing immunity against SARS-CoV-2, we generated OMVs decorated with polypeptides derived from the RBM of the ancestral strain. The RBM polypeptides were fused to FhuD2, a *S. aureus* lipoprotein shown to efficiently deliver heterologous protein domains to the *E. coli* outer membrane and to the vesicular compartment^7^.

To this end, the nucleotide sequence coding for the strands β5 (RBM_438-462_) and β6 (RBM_467-509_) or a combination of the two (RBM_438-509_) were fused to the 3’ end of the sequence encoding FhuD2 in plasmid pET-FhuD2^8^, thus generating three FhuD2-RBM fusion proteins (Figure 1B). The resulting plasmids were used to transform *E. coli BL21(DE3)Δ60* strain, a hyper-vesiculating *E. coli* BL21(DE3) derivative recently created in our laboratories^7^, thus creating the three recombinant strains *E. coliΔ60(*pET-FhuD2-RBM_438-462_), *E. coliΔ60(*pET-FhuD2-RBM_467-509_) and *E. coliΔ60(*pET-FhuD2-RBM_438-509_). From the culture supernatant of each recombinant strain the OMVs were concentrated, purified and subjected to SDS-PAGE analysis. As shown in Figure 1C, protein species compatible with the predicted molecular weights of the corresponding fusion proteins were clearly visible and based on the intensity of the bands (comparable with the band of the carrier FhuD2), the addition of the SARS-CoV-2 RBM polypeptides was well tolerated and compatible with efficient transport into the bacterial vesicles.

### Immunogenicity of FhuD2-RBM-OMVs

**Figure 2.**
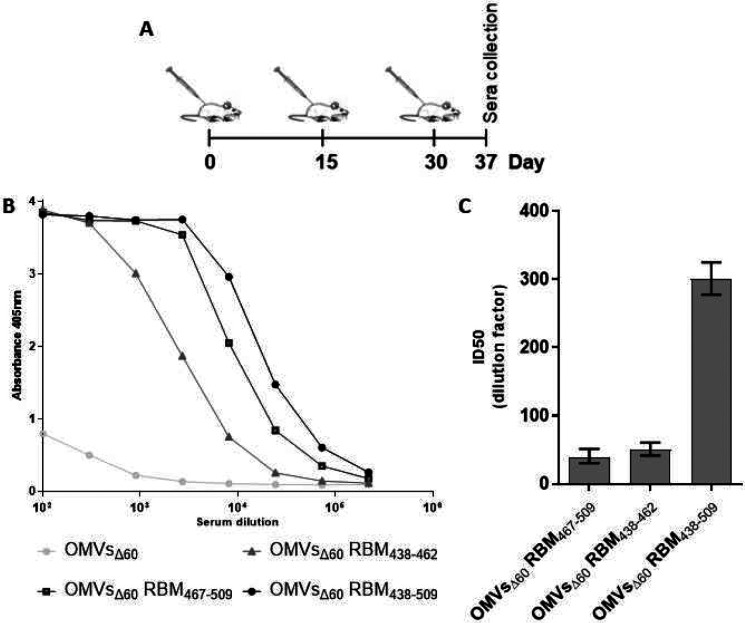
Mice immunized with OMVs decorated with SARS-CoV-2 RBM antigens produce neutralizing antibodies targeting the RBD (A) Diagram of the experimental setup of the immunization experiments. CD1 mice received three intraperitoneal immunizations on day 0, 14 and 28 with 10 μg of OMVs together with 2mg/ml Aluminium hydroxide. Sera were collected 10 days after the last immunization. As control, mice were immunized with non-engineered OMVs. (B) Antibody titers in sera pooled from each group of 5 mice, collected 10 days after the third immunization, measured by ELISA, using plates coated with the SARS-CoV-2 RBD. (C) Neutralization activity in pooled sera, measured with lentiviral vectors pseudotyped with SARS-CoV-2 spike from the ancestral isolate. In figure was reported the respective TCID50 values calculated with GraphPad. Plotted are the average values and the standard deviations from three determinations.

Having proven efficient association with OMVs, we next tested the capacity of FhuD2-RBM-OMVs to induce the production of antibodies capable of recognizing the RBM in the context of the SARS-CoV-2 Spike protein. To this aim, five CD1 mice were immunized with each construct three times at two-week intervals with 10 μg of each engineered OMV preparation combined with 2 mg/ml of Alum (Figure 2A) administered intraperitoneally. Blood samples were collected seven days after the third dose, pooled, and the titers of RBD-specific IgGs were detected by ELISA (Figure 2B) with plates coated with the spike RBD domain expressed and purified from HEK293T cells. Sera of animals immunized with the three different FhuD2-RBM-OMVs contained significant amounts of IgGs recognizing the RBD (Figure 2B), the highest titers being detected in sera from mice immunized with the fusions carrying either the β6 strand or the β5-β6 polypeptide.

Having demonstrated the presence of high-titers of anti-Spike antibodies with OMVs incorporating all three-fusion proteins, we next asked whether such antibodies could also neutralize virus infection *in vitro.* To this end, neutralization was assessed through a pseudovirus assay using a GFP-encoding lentiviral vector based on SIV and pseudotyped with the SARS-CoV-2 Spike protein. Sera derived from each group of mice immunized with the engineered OMVs were pooled, serially diluted and combined with the pseudotyped vectors before inoculation on Huh-7-ACE2 target cells.

The data shown in Figure 2C and S1 demonstrates that the sera collected from mice immunized with all three different OMVs incorporating the RBM fragments neutralize SARS-CoV-2 spike pseudotyped vectors. While the OMVs incorporating the single strands (RBM_438-462_ and RBM_467-509_) neutralized with similar potency with an ID50 of 1:50 and 1:39 respectively, OMV associated with the combination of the two beta strands (RBM_438-509_) induced a more powerful neutralization (ID50 of 1:301), indicating that both chains contribute and synergize to the elicitation of neutralizing antibodies. Interestingly, the neutralizing activity elicited by the RBM_ancestral_-OMVs in mice was of similar magnitude compared with the neutralization induced by the natural infection observed in convalescent patients which was measured using the same pseudovirus neutralization assay^23^. Therefore, in the subsequent experiments only the RBM_438-509_-OMVs were used and hereinafter named RBM_ancestral_-OMVs.

### Protective activity of RBM_ancestral_-OMVs in hACE2 transgenic mice

**Figure 3.**
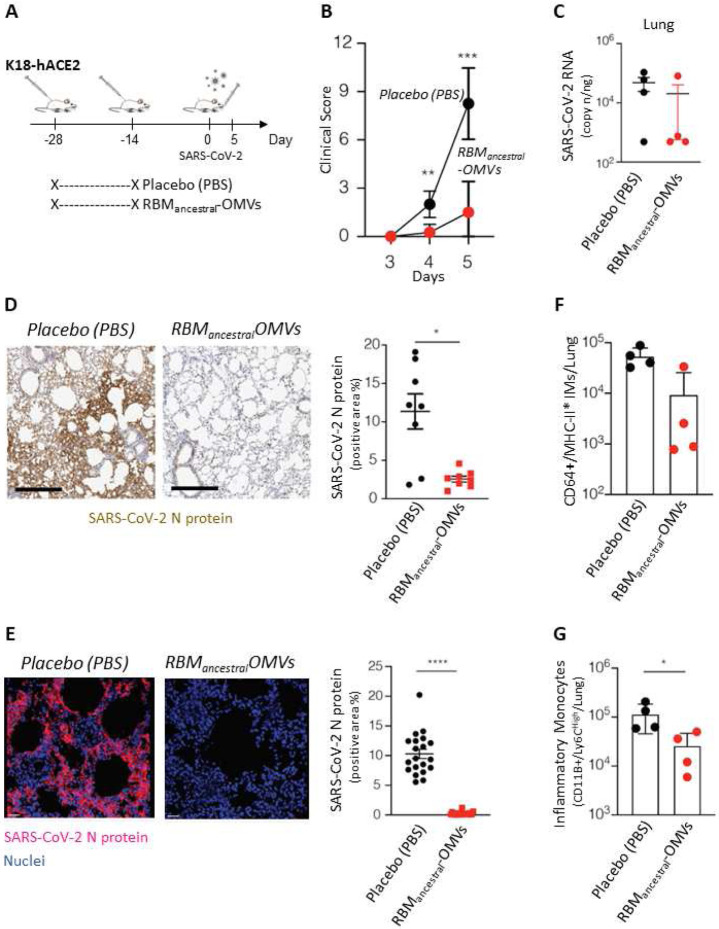
Immunization with RBM_ancestral_ decorated OMVs protects against SARS-CoV-2 challenge in hACE2 transgenic mice (A) Schematic representation of the experimental setup. K18-hACE2 mice (C57BL/6 background) received two intraperitoneal immunizations (at day −28 and −14) of 10 μg of OMVs vaccine (n = 4) or Placebo (PBS) (n = 4) prior to intranasal infection with 1×10^5^ TCID50 of SARS-CoV-2. Lung were collected and analyzed five days after SARS-CoV-2 infection. (B) Mice were observed daily for clinical symptoms to assess severity of disease based on respiration, coat condition, posture, social behavior, and palpebral aperture. (C) SARS-CoV-2 RNA in the lung was quantified by quantitative PCR with reverse transcription (RT–qPCR) 5 days after infection. (D) Representative immunohistochemical micrographs of lung sections 5 days post SARS-CoV-2 infection. N-SARS-CoV-2 expression is shown in brown. Scale bars, 300 μm. Right panel, quantification of N-SARS-CoV-2 signal, each dot represents a mouse. (E) Representative confocal immunofluorescence micrographs of lung sections from PBS-treated mice (left) or RBM_ancestral_-OMVs immunized mice (right) 5 days post SARS-CoV-2 infection. N-SARS-CoV-2 positive cells are depicted in purple and nuclei in blue. Scale bar represents 30 μm. Right panel, quantification of N-SARS-CoV-2 signal, each dot represents a different stack. (F) Absolute numbers of CD11b+/Ly6Chigh inflammatory monocytes in the lung of the indicated mice 5 days after SARS-Cov-2 infection. (G) Absolute numbers of CD64+/MHC-II+ inflammatory monocytes in the lung of the indicated mice. * p value < 0.05, ** p value < 0.01, *** p value < 0.001, **** p value < 0.0001

Having established that the RBM_ancestral_-OMVs are effective at inducing neutralizing immunity in mice, we selected the latter to explore the degree of protection induced *in vivo* against SARS-CoV-2 challenge. To this end, K18-hACE2 transgenic mice^24^ received two intraperitoneal immunizations (at day −28 and at day −14) of 10 μg of RBM_ancestral_-OMVs (*n* = 4) or Placebo (PBS) (*n* = 4) (Figure 3A). Fourteen days after the boost immunization, all mice were infected intranasally with 1 × 10^5^ TCID_50_ of SARS-CoV-2 (hCoV-19/Italy/LOM-UniSR-1/2020; GISAID Accession ID: EPI_ISL_413489) (Figure 3A). As expected^25^ beginning 4 days post infection (p.i.) PBS-treated K18-hACE2 transgenic mice infected with SARS-CoV-2 developed a severe disease, as assessed by monitoring respiration, coat condition, posture, social behaviour, and palpebral aperture^26^ (Figure 3B). By contrast, K18-hACE2 transgenic mice immunized with RBM_ancestral_-OMVs vaccine developed a much milder disease (Figure 3B). Viral RNA was measured, and infectious virus titrated from lungs of infected mice 5 days after challenge. Out of four vaccinated mice, only in one mouse viral RNA and replicating virus could be detected, indicating the ability of the vaccine to prevent virus replication in the respiratory tract (Figure 3C). Mirroring the relative abundance of viral RNA, the N protein was also readily detected by both immunohistochemistry and immunofluorescence microscopy in the lungs of PBS-treated mice while it was mostly absent in mice immunized with the RBM_ancestral_-OMVs (Figure 3D and E). The effective protection by the vaccine should also reflect the absence or attenuation of immune activation in response to the viral challenge. To assess the degree of inflammation triggered by the viral infection, we measured the number of inflammatory monocytes (CD11b+Ly6C high and CD64+) recruited in the lungs after infection. As shown in Figure 3F and G, in vaccinated mice a significantly lower number of inflammatory monocytes were recovered from the lungs of vaccinated mice compared to unvaccinated animals after viral challenge, correlating with the lower viral titers.

**Figure 4.**
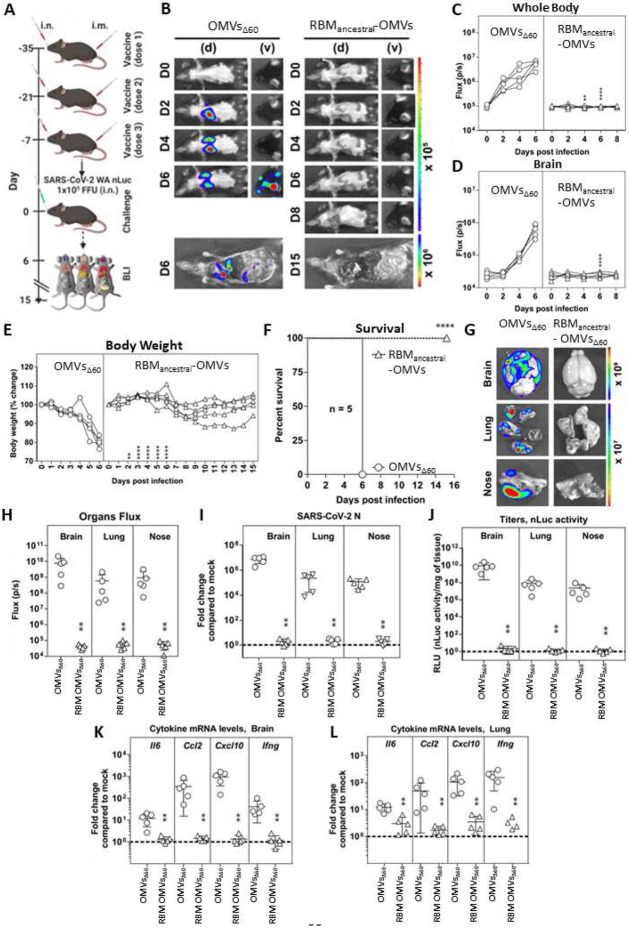
Immunization with RBM_ancestral_ decorated OMVs protects K18-hACE2 mice against lethal challenge with homologous SARS-CoV-2 ancestral strain (A) Experimental design to evaluate the immunization efficacy of OMVs decorated with RBM_ancestral_-OMVs in K18-hACE2 mice challenged with SARS-CoV-2-WA-nLuc (i.n., 1 x10^5^ FFU) Mice immunized with empty OMVs served as controls. Virus infection in mice were followed by non-invasive bioluminescence imaging (BLI) every 2 days from the start of infection. (B) Representative BLI images of SARS-CoV-2-WA-nLuc-infected mice in ventral (v) and dorsal (d) positions. Scale bars denote radiance (photons/sec/cm^2^/steradian). (C-D) Temporal quantification of nLuc signal as flux (photons/sec) computed non-invasively. (E) Temporal changes in mouse body weight with initial body weight set to 100. (F) Kaplan-Meier survival curves of mice (n = 5 per group) statistically compared by log-rank (Mantel-Cox) test for experiment as in A. (G, H) *Ex vivo* imaging of indicated organs and quantification of nLuc signal as flux(photons/sec) after necropsy for an experiment shown in A. (I) Fold change in SARS-CoV-2 nucleocapsid (N) mRNA expression in brain, lung and nose. (J) Viral loads (nLuc activity/mg) in indicated tissues measured after necropsy on Vero E6 cells as targets. (K, L) Fold change in indicated cytokine mRNA expression in brain and lung. The data were normalized to *Gapdh* mRNA expression in the same sample and that in non-infected mice after necropsy. Viral loads (I, J) and inflammatory cytokine profile (K, L) were determined at the time of death at 6 dpi or 15 dpi for surviving mice after necropsy. Each curve in C-E and each data point in H-L represents an individual mouse. Grouped data in (C-E) were analyzed by 2-way ANOVA followed by Sidak’s multiple comparison tests. The data in (H-L) was analyzed by t-test followed by non-parametric Mann-Whitney test *, p < 0.05; **, p < 0.01; ***, p < 0.001; ****, p < 0.0001; Mean values ± SD are depicted.

To further confirm the protective efficacy of RBM_ancestral_-OMVs we used a bioluminescence imaging (BLI) to monitor the impact of vaccination on replication and spread of reporter SARS-CoV-2 WA-nanoluc luciferase (nLuc) in K18-hACE2 mice. K18-hACE2 mice are extremely sensitive to SARS-CoV-2-induced mortality where virus spreads systemically to distal tissues such as brain, gastrointestinal tract, and testis in addition to nasal cavity and lung ^27^. Hence in these set of experimental evaluation, we immunized K18-hACE2 mice both intranasally and intramuscularly to elicit mucosal and peripheral immunity respectively with either RBM_ancestral_ decorated OMVs or “empty” OMVs_Δ60_ (Figure 4A). Non-invasive longitudinal BLI followed by quantification of nLuc signals in the whole body and brain, revealed that virus replicated unabatedly and reached the brain by 4 dpi in mice immunized with empty OMVs (Figure 4B-D). In contrast, nLuc signals were undetectable in RBM_ancestral_-OMVs immunized mice suggesting effective inhibition of SARS-CoV-2 replication. In accordance with the BLI, mice immunized with empty OMVs experienced gradual weight loss and succumbed to infection by 6 dpi, whereas those immunized with RBM_ancestral_-OMV experienced only mild weight loss (9-14 days) before recovery and demonstrated 100% survival (Figure 4E, F). nLuc signals measured after necropsy in isolated target organs (lung, brain, and nose) corresponded to viral loads (N mRNA expression, nLuc activity) and indicated significant inhibition of virus titres in Rac_kstraw_ OMV-immunized mice compared to empty OMV immunized controls (Figure 4G-J). Analyses of inflammatory cytokine mRNA expression (*Il-6, Ccl2, Cxcl10, and Ifnγ*) in target organs also revealed a 10- to 1,000-fold reduction in RBM_ancestral_-OMVs vaccinated mice compared to empty OMVs (Figure 4K, L). These data indicate that vaccination with RBM_ancestral_-OMVs protected mice against lethal SARS-CoV-2 challenge by preventing both viral replication and ensuing inflammatory cytokine response.

### Engineering and immunogenicity of OMVs decorated with the RBM from omicron BA.1 variant

**Figure 5.**
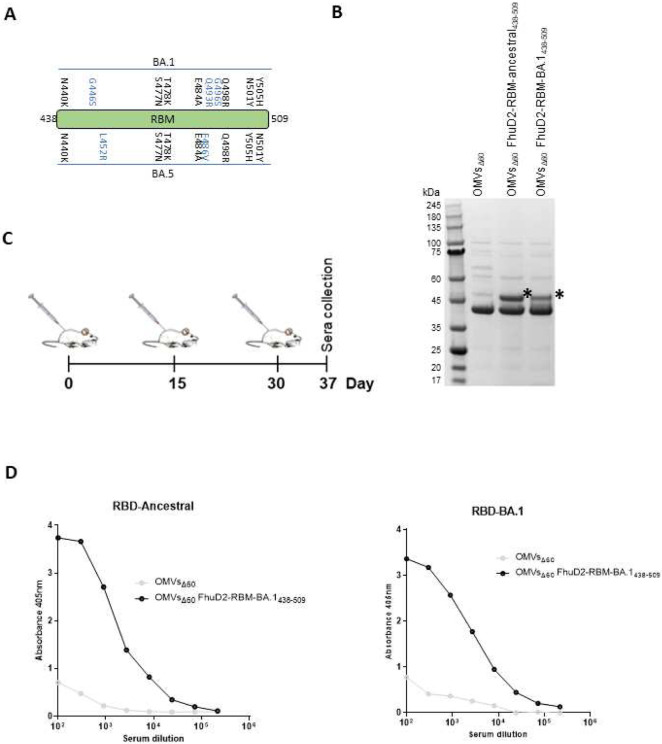
Production and immunogenicity of OMVs carrying SARS-CoV-2 RBM antigens derived from the omicron BA.1 isolate of SARS-CoV-2 (A) schematic of the residues found in micron BA.1 and BA.5 RBM which differ from to the reference ancestral strain sequence. Residues in blu are uniquely present in BA.1 and BA.5 strains. (B) SDS-PAGE of purified OMVs decorated with RBM from ancestral and omicron BA.1 strains generated by BL21(DE3)*Δ60* cultures expressing pET21-FhuD2-RBM_438-462_ and pACYC- FhuD2-RBM-BA.1_438-462_ plasmids, respectively or the control empty pET21 plasmid. (D) Diagram of the experimental setup of the immunization experiments. CD1 mice received three intraperitoneal immunizations on day 0, 15 and 30 with 10 μg of OMVs together with 2mg/ml Aluminium hydroxide. Sera were collected 7 days after the last immunization. As control, mice were immunized with non-engineered OMVs. (D) Antibody titers in sera pooled from each group of 5 mice, collected 10 days after the third immunization, measured by ELISA, using plates coated with the SARS-CoV-2 RBD from the ancestral strain (left) and omicron BA.1 (right).

In order to test the adaptability of the OMV platform, we engineered the vesicles with the combination of β5 and β6 strands (RBM) from Omicron BA.1 variant, which carry 10 amino acid differences with respect to the ancestral RBM (Figure 5A). As shown in Figure 5B, RBM BA.1_438-509_ fused to FhuD2 were expressed in *E. coli BL21(DE3)Δ60* and efficiently compartmentalized in the OMVs.

Next, we asked the question whether RBM_BA.1_-OMVs could elicit antibodies capable of recognizing the corresponding purified RBD. Mice were immunized with the engineered OMVs following the time schedule described in Figure 5C and sera were collected seven days after the last injection. Antibody titers were assessed by ELISA coating the plates with either purified RBD_ancestral_ or purified RBD_BA.1_. As a control, the pool of sera from mice immunized with RBM_ancestral_-OMVs was also tested against the two RBDs. As shown in Figure 5C, RBM_BA.1_-OMVs elicited elevated IgG titers against both the homologous RBD and ancestral-RBD. By contrast, the RBM_ancestral_-OMVs serum could efficiently recognize the heterologous RBD_BA.1_ at similar titers to ancestral-RBD.

### RBM_ancestral_-OMVs elicit cross-neutralizing antibodies

**Figure 6.**
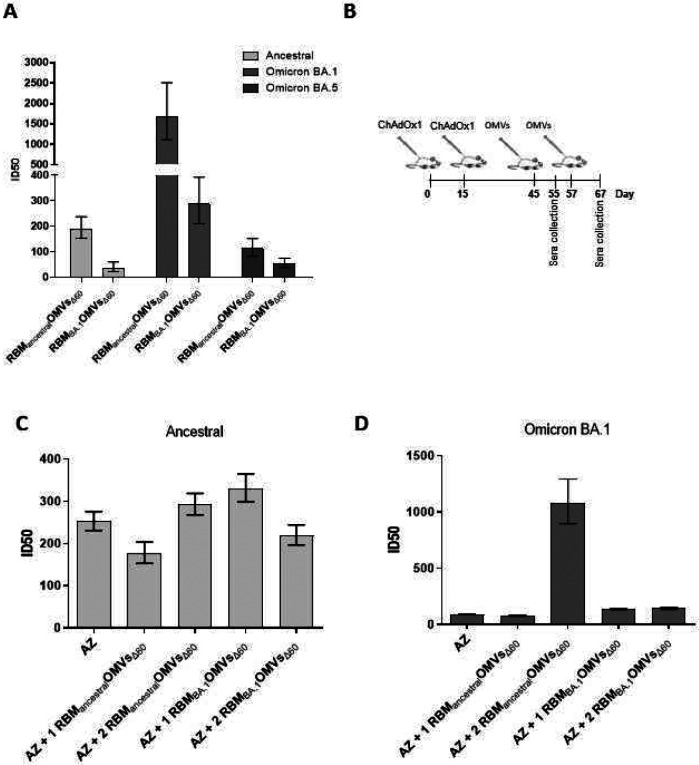
Ability of OMVs decorated with RBM antigens derived from the ancestral and omicron BA.1 isolates to boost immunity previously elicited by ChAdOx1 (A) neutralization activity in pooled sera from animals immunized with OMVs decorated with RBM derived from the ancestral strain or the omicron BA.1 variant. Neutralization was measured with lentiviral vectors pseudotyped with SARS-CoV-2 spike from the ancestral isolate, omicron BA.1 and BA.5 variants, plated on Huh-7 cells. Shown are ID50 values calculated from the curves shown in Figure S2 with GraphPad. Error bars represent the standard deviations from three determinations. (B) Diagram of the experimental setup of the immunization experiments. CD1 mice received two intraperitoneal immunizations with ChAdOx1 (AZ) on day 0 and 15 and two further immunization with OMVs decorated either with RBM from the ancestral or omicron BA.1 strains. Sera were collected prior to the second OMVs immunization or 7 days after the last immunization. As control, mice were immunized with non-engineered OMVs. (C and D) Neutralization activity in sera from animals immunized following the experimental setup in B, measured with lentiviral vectors pseudotyped with SARS-CoV-2 spike from the ancestral isolate and the omicron BA.1 avariant, plated on Huh-7 cells. Shown are ID50 values calculated from the curves shown in Figure S3 with GraphPad. Error bars represent the standard deviations from three determinations

We next tested whether the anti-RBD_BA.1_ antibodies elicited by RBM_ancestral_-OMVs could also neutralize SARS-CoV-2 pseudotyped vectors expressing the omicron BA.1 and BA.5 variants spike proteins. This would be an unexpected result, considering that patients infected with the original ancestral strain and vaccinees that received Spike_ancestral_-based vaccinees are poorly protected against the Omicron variants. In addition, most monoclonal antibodies^28^, which potently neutralize the ancestral strain by binding to the RBM region, are almost entirely ineffective against Omicron^29,30^. We, therefore, tested the sera from mice immunized with RBM_ancestral_-OMVs and RBM_BA.1_-OMVs for their *in vitro* neutralization capacity against the ancestral, the Omicron BA.1 and Omicron BA.5 pseudoviruses. As shown in Figure 6A and S2, sera from RBM_BA.1_-OMVs immunized mice neutralized Omicron BA.1 pseudovirus with ID 50 of 1:300. In contrast, the same sera were ineffective against ancestral and Omicron BA.5 (ID50<50), suggesting that the neutralizing antibodies elicited by omicron BA.1 RBM are highly variant-selective and therefore poorly cross-neutralizing. Such neutralization behavior diverges from the binding activity of the same sera, observed with ELISA (Figure 5B), which indicates that antibodies from RBM_BA.1_-OMVs immunized mice bind the RBD derived from ancestral and Omicron BA.1 with equal efficiencies. Therefore, despite binding the RBD, antibodies elicited by RBM_BA.1_ do not neutralize the ancestral pseudoviruses. In contrast, sera from animals that received RBM_ancestral_-OMVs displayed the ability to cross-neutralize all three SARS-CoV-2 variants tested, though with surprisingly different efficiencies. While ancestral and omicron BA.5 pseudoviruses were neutralized with ID50 ranging from 1:100 to 1:200, omicron BA.1 pseudoviruses were neutralized with an average ID50 of 1:1500 (Figure 6A). Therefore, omicron RBM_ancestral_ displayed on OMVs has a greater ability than omicron RBM_BA.1_ to elicit broadly neutralizing antibodies, for which omicron BA.1 pseudoviruses are extraordinarily sensitive. Altogether, the results of these experiments point to RBM_ancestral_-OMVs as a potential anti-COVID19 pan vaccine.

Considering that a large proportion of the worldwide population has been either infected with SARS-CoV-2 or has been vaccinated with ancestral-based vaccines, we investigated the immune responses elicited by RBM-OMVs in animals that had previously received the AstraZeneca ChAdOx1 nCoV-19 vaccine (AZ). Animals were given two doses of ChAdOx1 nCoV-19 at two-week interval, and after one month from the second dose, mice were vaccinated twice with RBM_ancestral_-OMVs. Sera were collected ten days after the ChAdOx1 nCoV-19 vaccination and ten days after the first and the second dose of the OMV-based vaccine (Figures 6B and C and S3). All sera were tested for their capacity to neutralize both the ancestral strain and the Omicron BA.1 variant using the *in vitro* pseudovirus assay. As expected, the pool of sera from ChAdOx1 nCoV-19-immunized mice effectively neutralized the homologous pseudovirus (Figure 6B, ID50 ranging between 1:200 and 1:320) while showing poor neutralization capacity against the Omicron variant (Figure 6C, ID50 <100). A similar neutralization pattern was observed in sera from animals after the administration of a single dose of RBM_ancestral_-OMVs. By contrast, while not further increasing to the neutralization capacity already elicited by ChAdOx1 against ancestral pseudoviruses (Figure 6C), two doses of RBM_ancestral_-OMVs dramatically increased the neutralization titers against BA.1 pseudoviruses (ID50 1:1500), mirroring the effect of the immunization with RBM_ancestral_-OMVs alone (Figure 6A). These data suggest that ChAdOx1 nCoV-19 vaccine elicits neutralizing antibodies which differ from those induced by RBM-OMVs immunization and therefore ChAdOx1 nCoV-19-immunized mice behaved like “naive” animals with respect to OMVs vaccination.

## Discussion

We believe that this work delivers a few key messages.

First of all, we demonstrate for the first time that OMVs can be an amenable platform for the development of an effective SARS-CoV-2 vaccine. While properly folded eukaryotic glycoproteins can normally only be expressed in a eukaryotic expression system, the crucial portion of the Spike RBM, which directly contacts ACE2, can be efficiently incorporated into OMVs while maintaining a close-to-natural conformation. An effective immunity is elicited in animals vaccinated with RBM_ancestral_-OMVs, with production of neutralizing antibodies at levels sufficient to fully protect hACE2-transgenic mice from infection. In our view this is an interesting result, particularly in consideration of the fact that OMVs are extremely easy to produce. The separation of the biomass from the culture supernatant and an ultrafiltration step to concentrate OMVs and eliminate contaminants is essentially all is needed for vaccine production^14^. The process is amenable to large-scale production and can be easily transferred to different sites for expanding vaccine production. Moreover, the production yields make the vaccine costs particularly affordable. Under laboratory conditions, we reproducibly obtain more than 5,000 OMV-based vaccine doses/liter of culture. Practically speaking, it means that using a 1.000-litre fermentation unit associated with a tangential flow ultrafiltration device is sufficient to provide 5 million doses of vaccine/week at costs that are expected to be well below 1 USD/dose.

The second message is that the intranasal delivery of the RBM_ancestral_-OMV vaccine can potentially be particularly effective in preventing infection in the upper respiratory tract and viral dissemination in different organs and tissues, including the brain. This can be at least partially inferred by the two sets of animal experiments described in this work. In the first set of experiments, we immunized hACE2 transgenic mice systemically and subsequently we challenged mice intranasally with the ancestral strain. Three out of four animals survived, and viruses were not detected, using both immunohistochemistry and PCR, in the lungs of the protected mice. However, the virus could be found in the brain tissue of both protected and mock-immunized mice. In the second set of experiments, the RBM_ancestral_-OMV vaccine was given both systemically and intranasally before challenge. Following this immunization schedule, the five immunized animals were completely protected, viruses could not be detected in the lung, brain and nose of the animals, and protection strongly correlated with the absence of inflammatory cytokines in the analyzed tissues. Therefore, although we have not tested yet the intranasal immunization alone, the data suggest that mucosal delivery contributes substantially to overall protection.

The third important message of our work is that the RBM_ancestral_-OMVs vaccine elicits cross-neutralizing antibodies against different VOCs. The OMVs platform is flexible enough to be rapidly adapted to cope with new emerging variants. In addition to the RBM of the ancestral and BA.1 strain, we have successfully engineered our OMVs with the RBM from the P1 and B.1.617 isolates (not shown). All engineered OMVs elicited good levels of antibodies, which effectively neutralized the homologous pseudoviruses *in vitro.* However, quite surprisingly, we found that the antibodies induced by the RBM_ancestral_-OMVs vaccine provided robust cross-protection, as judged by the in vitro neutralization assay, against the most recent Omicron variants. Considering that the vaccines and the neutralizing monoclonal antibodies based on the ancestral Spike protein are known to be poorly effective against Omicron variants our results are of particular interest. One explanation for such unexpected crossreactivity could be that when expressed on OMVs, the RBM of the ancestral strain, but not the RBM of the BA.1 strain, present otherwise poorly immunogenic epitopes which induce antibodies which bind to conserved structural regions of the RBD involved in the interaction with the ACE2 receptor. It would be of particular interest to isolate the B cells from the RBM_ancestral_-OMVs immunized mice and characterize these putative new classes of antibodies. The existence of a new family of neutralized antibodies would be of particular interest from a vaccination standpoint. Nowadays, most of the worldwide population has been either infected by SARS-CoV-2 or vaccinated. Therefore, the immunization with the RBM_ancestral_-OMVs vaccine should synergize with the natural immunity of the vaccinees, thus making the vaccination particularly effective.

In conclusion, our data strongly suggest that the RBM_ancestral_-OMVs has the potential to become a vaccine with pan-SARS-CoV-2 efficacy that, thanks to the ease of manufacturing and low production costs, could be produced and broadly distributed in low-income countries.

## Materials and Methods

### Engineering BL21(DE3) E. coli strains with SARS-CoV-2 neutralizing epitopes

The pET21-FhuD2 plasmid carrying the *Staphylococcus aureus* Ferric hydroxamate receptor 2 (FhuD2) was fused to one copy of RBM_438-462_, RBM_467-509_ and RBM_438-509_ SARS-CoV-2 epitope, respectively. The three plasmids were assembled using the PIPE method as described in ^31^. Briefly, pET21-FhuD2 was linearized by PCR, using FhuD2-v-R and pETV-F primers (Table 1). In parallel, the synthetic DNA encoding the RBM of SARS-CoV-2 was synthesized by GeneArt (Thermo Fisher Scientific, Waltham, MA, USA) and used as template for the amplification of the three epitopes. More in detail, RBM_438-462_ and RBM_467-509_ were amplified by PCR with the forward 2-F and 1-F and the reverse 2-R and 1-R primers, respectively (Table 2). The PCR products and the linearized plasmid were mixed together and used to transform *E. coli* HK100 strain.

The RBM_438-509_, the combination of the two epitopes, was assembled with two steps of PCR in succession. In the first step two different fragments carrying an overlapping sequence were amplified with 2-F/R-1 and F-1/1-R primers. In the second step the two fragments were eluted from Agarose gel, mixed together and used as template for a final PCR reaction with the primers 2-F and 1-R. This final product and the linearized plasmid were mixed together and used to transform *E. coli* HK100 strain. ,

The RBD-BA.1_438-509_ gene, encoding for a variant of the RBD carrying the mutations shown in Figure 5A, was synthesized by GeneArt (Thermo Fisher Scientific, Waltham, MA, USA) and used as template for the amplification of the RBM-BA.1_438-509_ using the CoSASta-F and CoSASta-R primers. The low copy number pACYC plasmid was linearized using the couple of primers FhuD2-v-R and PACYC-F The PCR product and the linearized plasmid were mixed together and used to transform *E. coli* HK100 strain.

To confirm the correct gene fusions, plasmids were sequenced (Eurofins, Ebersberg, Germany, EU) and *E. coli* BL21(DE3)Δ60 strain was transformed with pET21-FhuD2-RBM_438-462_, pET21-FhuD2-RBM_467-509_, pET21-FhuD2-RBM_438-509_ and pACYC-FhuD2-RBM-BA.1_438-509_ plasmids and the derived recombinant strains were used for the production of engineered FhuD2-RBM_438-462_, FhuD2-RBM_467-509_, FhuD2-RBM_438-509_ (RBM_ancestral_-OMVs) and FhuD2-RBM-BA.1_438-509_ (RBM_BA.1_-OMVs) OMVs, respectively.

### OMV purification

OMVs from BL21(DE3)*Δ60*-pET-FhuD2-RBM_438-509_, BL21(DE3)*Δ6*0-pET-FhuD2-RBM_438-462_, BL21(DE3)*Δ60*-pET-FhuD2-RBM_467-509_ and BL21(DE3)*Δ60*-pACYC-FhuD2-RBMBA.1_438-509_ were purified in an EZ control bioreactor (Applikon Biotechnology, Schiedam, Netherlands) as previously described^7^. Cultures were started at an OD_600_ of 0.1 and grown in LB medium at 30°C, pH 6.8 (±0.2), dO_2_ > 30%, 280–500 rpm until OD_600_=0.5, then the temperature was lowered to 25°C. The expression of the recombinant protein was induced when the culture reached an OD_600_ of 1 with 0.1 mM IPTG and a feed made of 50 mg/l ampicillin, 15 g/l glycerol, 0.25 g/l MgSO_4_ was added. The fermentation was carried out until the end of the exponential phase at 25°C. OMVs were then purified and quantified as previously described^7^. Culture supernatants were separated from biomass by centrifugation at 4000g for 20 minutes. After filtration through a 0.22-μm pore size filter (Millipore, Burlington, Massachusetts, USA), OMVs were isolated, concentrated and diafiltrated from the supernatants using Tangential Flow Filtration (TFF) with a Cytiva Äkta Flux system. OMVs were quantified using DC protein assay (Bio-Rad, Hercules, California. USA).

### SDS-PAGE

20 μg (protein content) were resuspended in sodium dodecyl sulfate-polyacrylamide gel electrophoresis (SDS-PAGE) Laemmli buffer and heated at 98°C for 10’. Proteins were separated using Any kD^™^ Criterion^™^ TGX Stain-Free^™^ Protein Gel (BioRad) in TrisGlicyne buffer (BioRad) and proteins were revealed by Coomassie Blue staining.

### Studies with CD1 mice

Mice were monitored twice per day to evaluate early signs of pain and distress, such as respiration rate, posture, and loss of weight (more than 20%) according to humane endpoints. Animals showing such conditions were anesthetized and subsequently sacrificed in accordance with experimental protocols, which were reviewed and approved by the Animal Ethical Committee of The University of Trento and the Italian Ministry of Health. Five-week old CD1 female mice were immunized intraperitoneally (i.p.) on day 0, 14 and 28 with 10 μg of OMVs together with 2mg/ml Aluminium hydroxide in a final volume of 200 μl. Sera were collected 10 days after the last immunization. Alternatively, mice received intramuscularly (i.m.) 50 μl of AstraZeneca ChAdOx1 nCoV-19 vaccine, consisting in roughly 2.5x10^7^ infectious units.

### Studies with the ACE2 Mouse model

B6.Cg-Tg(K18-ACE2)^2Prlmn/^J mice^1^ were purchased from The Jackson Laboratory. Mice were housed under specific pathogen-free conditions and heterozygous mice were used at 8-10 weeks of age. All experimental animal procedures were approved by the Institutional Animal Committee of the San Raffaele Scientific Institute and all infectious work was performed in designed BSL-3 workspaces. The hCoV-19/Italy/LOM-UniSR-1/2020 (EPI_ISL_413489) isolate of SARS-CoV-2 was obtained from the Unit of Microbiology and Virology of San Raffaele Scientific Institute and grown in Vero E6 cells. K18-hACE2 transgenic mice were immunized intraperitoneally with 10μl vaccine or PBS twice, 14 days apart. Virus infection was performed via intranasal administration of 1 x 10^5^ TCID_50_ SARS-CoV-2 per mouse. Tissues homogenates were prepared by homogenizing perfused lung using gentleMACS Octo Dissociator (Miltenyibiotec, #130-096-427) in M tubes (#130-093-335) containing 1 ml of DMEM. Samples were homogenized for three times with program m_Lung_01_02 (34 seconds, 164 rpm). The homogenates were centrifuged at 3’500 rpm for 5 minutes at 4°C. The supernatant was collected and stored at −80°C for viral isolation and viral load detection. Viral titers were calculated by 50% tissue culture infectious dose (TCID_50_). Briefly, Vero E6 cells were seeded at a density of 1.5 × 10^4^ cells per well in flat-bottom 96-well tissue-culture plates. The following day, 2-fold dilutions of the homogenized tissue were applied to confluent cells and incubated 1 h at 37°C. Then, cells were washed with phosphate-buffered saline (PBS) and incubated for 72 h at 37°C in DMEM 2% FBS. Cells were fixed with 4% paraformaldehyde for 20 min and stained with 0.05% (wt/vol) crystal violet in 20% methanol.

### RNA extraction and qPCR

Tissues homogenates were prepared by homogenizing perfused lung using gentleMACS dissociator (Miltenyibiotec, #130-096-427) with program RNA_02 in M tubes (#130-096-335) in 1 ml of Trizol (Invitrogen, #15596018). The homogenates were centrifuged at 2000 g for 1 min at 4°C and the supernatant was collected. RNA extraction was performed by combining phenol/guanidine-based lyisis with silica membrane-based purification. Briefly, 100 μl of Chloroform were added in 500 ml of homogenized sample and total RNA was extracted using ReliaPrep^™^ RNA Tissue Miniprep column (Promega, Cat #Z6111). Total RNA was isolated according to the manufacturer’s instructions. qPCR was performed using TaqMan Fast virus 1 Step PCR Master Mix (Lifetechnologies #4444434), standard curve was drawn with 2019_nCOV_N Positive control (IDT#10006625), primer used are: 2019-nCoV_N1- Forward Primer (5’-GAC CCC AAA ATC AGC GAA AT-3’), 2019-nCoV_N1-Reverse Primer (5’-TCT GGT TAC TGC CAG TTG AAT CTG-3’) 2019-nCoV_N1-Probe (5’-FAM-ACC CCG CAT TAC GTT TGG TGG ACC-BHQ1-3’) (Centers for Disease Control and Prevention (CDC) Atlanta, GA 30333). All experiments were performed in duplicate.

### Cell Isolation and Flow Cytometry

Mice were euthanized by cervical dislocation. Lungs were perfused through the right ventricle with PBS at the time of autopsy. Lung tissue was digested in RPMI 1640 containing 3.2 mg/ml Collagenase IV (Sigma, #C5138) and 25 U/ml DNAse I (Sigma, #D4263) for 30 minutes at 37°C. Homogenized lungs were passed through 70 μm nylon mesh to obtain a single cell suspension. Cells were resuspended in 36% percoll solution (Sigma #P4937) and centrifuged for 20 minutes at 2000 rpm (light acceleration and low brake). The remaining red blood cells were removed with ACK lysis. Cell viability was assessed by staining with Viobility^™^ 405/520 fixable dye (Miltenyi, Cat #130-109-814). Antibodies (Abs) used are indicated in Table 3. Flow cytometry analysis were performed on a BD FACSymphony A5 SORP and analyzed with FlowJo software (Treestar).

### Confocal immunofluorescence histology and histochemistry.

Lungs of infected mice were collected and fixed in 4% paraformaldehyde (PFA). Samples were then dehydrated in 30% sucrose prior to embedding in OCT freezing media (Bio-Optica). Twenty micrometer sections were cut on a CM1520 cryostat (Leica) and adhered to Superfrost Plus slides (Thermo Scientific). Sections were then permeabilized and blocked in PBS containing 0.3% Triton X-100 (Sigma-Aldrich) and 5% FBS followed by staining in PBS containing 0.3% Triton X-100 and 1% FBS. Slides were stained for SARS-CoV-2 nucleocapsid (GeneTex) for 1h RT. Then, slides were stained with Alexa Fluor 568 Goat Anti-Rabbit antibody for 2h RT. All slides were analyzed by confocal fluorescence microscopy (Leica TCS SP5 Laser Scanning Confocal). For N-SARS-CoV-2 immunohistochemistry, mice were perfused with PBS and lung were collected in Zn-formalin and transferred into 70% ethanol 24 h later. Tissue was then processed, embedded in paraffin and automatically stained for SARS-CoV-2 (2019-nCoV) Nucleocapsid Antibody (SINO BIO, 40143-R019) through LEICA BOND RX 1h RT and developed with Bond Polymer Refine Detection (Leica, DS9800). Brightfield images were acquired through an Aperio Scanscope System CS2 microscope and an ImageScope program (Leica Biosystem) following the manufacturer’s instructions. In both immunofluorescence and histochemistry, N-SARS-CoV-2 percentage of positive area was determined by QuPath (Quantitative Pathology & Bioimage Analysis) software.

### Clinical score

Mice were observed daily for clinical symptoms. Disease severity was scored as follows:

**Table:** 

Respiration	slight alteration	1
	moderate alteration	2
	marked alteration	3
Coat condition	slight piloerection	1
	moderate piloerection	2
	marked piloerection	3
Posture	hunched posture	1
Social behavior	reduced interaction with animals	1
	marked reduced interaction with animals	2
	lack of grooming	2
	passive + tremors	3
Palpebral aperture	Open	0
	half closed	2
	sunken and half closed	3

### Studies with hACE2 transgenic B6 mice using bioluminescent viruses.

All animals were maintained in the (SPF-free) barrier facility of the Yale University Animal Resource Centre (YARC) within a 14:10 light: dark cycle. Breeding population of mice and infected animals are maintained in separate rooms. All SARS-CoV-2-infected animals were housed in animal room under BSL3 containment. Cages, animal waste, bedding, and animal carcasses were disposed and decontaminated following the guidelines of Environmental Health Services at Yale. All replication competent virus-infected animals were handled under ABSL3 conditions with personnel’s donning pressurized air purified respirators (PAPR), double gloves, shoe covers, sleeve covers and disposable gowns. All experiments described here were approved by Institutional Animal Care and Use Committees (IACUC) as well as SOPs approved by Institutional Environmental Health and Biosafety committee. hACE2 transgenic B6 mice (heterozygous) were obtained from Jackson Laboratory. 6–8-week-old male and female mice were used for all the experiments. The heterozygous mice were crossed and genotyped to select heterozygous mice for experiments by using the primer sets recommended by Jackson Laboratory. Each cohort size was n = 5 to allow statistical testing. The number of animals (n = 4–8 per cohort) needed to achieve statistically significant results were calculated based on a priori power analysis. We calculated power and sample sizes required based on data from pilot experiments and previous studies^27,32,33^ Animals with sex- and age-matched littermates were included randomly in the experiments. No animals were excluded due to illness after the experiments. At the time of experimentation, care was taken to include equal numbers of male and female mice whenever possible to ensure that sex of the animals does not constitute a biological variable during analysis.

### OMV vaccination and SARS-CoV-2 infection

For all bioluminescence imaging-based *in vivo* experiments shown in Figure 4, the 6 to 8 weeks male and female mice (n=2 or 3 each) were immunized with empty or RBM_ancestral_-OMVs both intramuscularly (i.m., 50 μL per mouse; mixed with 2 mg/ml aluminium hydroxide on day of immunization, day 0) and intranasally (i.n., 25 μL, without any adjuvant) The animals were then boosted on day 14 and day 28 both i.m and i.n in a similar manner as vaccination. On day 35, the mice were intranasally challenged with 1x10^5^ FFU SARS-CoV-2_WA1_nLuc in 25-30 μL volume under anesthesia (0.5 - 5 % isoflurane delivered using precision Dräger vaporizer with oxygen flow rate of 1 L/min). The starting body weight was set to 100 %. For survival experiments, mice were monitored every 8-12 h starting six days after virus challenge. Lethargic and moribund mice or mice that had lost more than 20 % of their body weight were sacrificed and considered to have succumbed to infection for Kaplan-Meier survival plots. Mice were considered to have recovered if they gained back all the lost weight (experimental endpoint).

### Bioluminescence Imaging (BLI) of SARS-CoV-2 infection

All standard operating procedures and protocols for IVIS imaging of SARS-CoV-2-infected animals under ABSL-3 conditions were approved by IACUC, IBSCYU and Yale Animal Research Center (YARC). All the imaging was carried out using IVIS Spectrum^®^ (PerkinElmer) in XIC-3 animal isolation chamber (PerkinElmer) that provided biological isolation of anesthetized mice or individual organs during the imaging procedure. All mice were anesthetized via isoflurane inhalation (3 - 5 % isoflurane, oxygen flow rate of 1.5 L/min) prior and during BLI using the XGI-8 Gas Anesthesia System. Prior to imaging, 100 μL of nanoluc luciferase (nLuc) substrate, furimazine (NanoGlo^™^, Promega, Madison, WI) diluted 1:40 in endotoxin-free PBS was retroorbitally administered to mice under anesthesia. The mice were then placed into XIC-3 animal isolation chamber (PerkinElmer) pre-saturated with isothesia and oxygen mix. The mice were imaged in both dorsal and ventral position at indicated days post infection. The animals were then imaged again after euthanasia and necropsy by spreading additional 200 μL of substrate on to exposed intact organs. Infected areas identified by carrying out whole-body imaging after necropsy were isolated, washed in PBS to remove residual blood and placed onto a clear plastic plate. Additional droplets of furimazine in PBS (1:40) were added to organs and soaked in substrate for 1-2 min before BLI. Images were acquired and analyzed with Living Image v4.7.3 *in vivo* software package (Perkin Elmer Inc). Image acquisition exposures were set to auto, with imaging parameter preferences set in order of exposure time, binning, and f/stop, respectively. Images were acquired with luminescent f/stop of 2, photographic f/stop of 8. Binning was set to medium. Comparative images were compiled and batch-processed using the image browser with collective luminescent scales. Photon flux was measured as luminescent radiance (p/sec/cm^2^/sr). During luminescent threshold selection for image display, luminescent signals were regarded as background when minimum threshold setting resulted in displayed radiance above non-tissue-containing or known uninfected regions.

### Focus forming assay

Titers of virus stocks was determined by standard plaque assay. Briefly, the 4 x 10^5^ Vero-E6 cells were seeded on 12-well plate. 24 h later, the cells were infected with 200 μL of serially diluted virus stock. After 1 hour, the cells were overlayed with 1ml of pre-warmed 0.6% Avicel (RC-581 FMC BioPolymer) made in complete RPMI medium. Plaques were resolved at 48 h post infection by fixing in 10 % paraformaldehyde for 15 min followed by staining for 20 min with 0.2 % crystal violet made in 20 % ethanol. Plates were rinsed in water to visualize plaques.

### Measurement of viral burden

Indicated organs (nasal cavity, brain, lungs) from infected or uninfected mice were collected, weighed, and homogenized in 1 mL of serum free RPMI media containing penicillin-streptomycin and homogenized in 2 mL tube containing 1.5 mm Zirconium beads with BeadBug 6 homogenizer (Benchmark Scientific, TEquipment Inc). Virus titers were measured using three highly correlative methods. Frist, the total RNA was extracted from homogenized tissues using RNeasy plus Mini kit (Qiagen Cat # 74136), reverse transcribed with iScript advanced cDNA kit (Bio-Rad Cat #1725036) followed by a SYBR Green Realtime PCR assay for determining copies of SARS-CoV-2 N gene RNA using primers SARS-CoV-2 N F: 5’-ATGCTGCAATCGTGCTACAA-3’ and SARS-CoV-2 N R: 5’-GACTGCCGCCTCTGCTC-3’. All our real-time PCR assays based on SYBR Green had a built-in melt-curve that were checked to ensure estimation of only specific PCR products and not false-positives. Second, we used Nanoluc activity as a shorter surrogate for plaque assay. Infected cells were washed with PBS and then lysed using 1X Passive lysis buffer. The lysates transferred into a 96-well solid white plate (Costar Inc) and nLuc activity was measured using Tristar multiwell Luminometer (Berthold Technology, Bad Wildbad, Germany) for 2.5 seconds by adding 20 μl of Nano-Glo^®^ substrate in nanoluc assay buffer (Promega Inc, WI, USA). Uninfected monolayer of Vero cells treated identically served as controls to determine basal luciferase activity to obtain normalized relative light units. The data were processed and plotted using GraphPad Prism 8 v8.4.3.

### Analyses of signature inflammatory cytokines mRNA expression

Brain and lung samples were collected from mice at the time of necropsy. Approximately, 20 mg of tissue was suspended in 500 μL of RLT lysis buffer, and RNA was extracted using RNeasy plus Mini kit (Qiagen Cat # 74136), reverse transcribed with iScript advanced cDNA kit (Bio-Rad Cat #1725036). To determine mRNA copy numbers of signature inflammatory cytokines, multiplex qPCR was conducted using iQ Multiplex Powermix (Bio Rad Cat # 1725848) and PrimePCR Probe Assay mouse primers FAM-GAPDH, HEX-IL6, TEX615-CCL2, Cy5-CXCL10, and Cy5.5-IFNgamma. The reaction plate was analyzed using CFX96 touch real time PCR detection system. Scan mode was set to all channels. The PCR conditions were 95 °C 2 min, 40 cycles of 95 °C for 10 s and 60 °C for 45 s, followed by a melting curve analysis to ensure that each primer pair resulted in amplification of a single PCR product. mRNA copy numbers of *Il6, Ccl2, Cxcl10 and Ifng* in the cDNA samples of infected mice were normalized to *Gapdh* mRNA with the formula ΔC_t_(target gene)=C_t_(target gene)-C_t_(*Gapdh*). The fold increase was determined using 2^−ΔCt^ method comparing treated mice to uninfected controls.

### Statistical analyses and software

Detailed information concerning the statistical methods used is provided in the figure legends. Flow data were collected using FlowJo Version 10.5.3 (Treestar). Statistical analyses were performed with GraphPad Prism software version 8 (GraphPad). *n* represents individual mice analyzed per experiments. Error bars indicate the standard error of the mean (SEM).

### Cell and Viruses

Vero E6 (CRL-1586, American Type Culture Collection (ATCC), were cultured at 37°C in RPMI supplemented with 10% fetal bovine serum (FBS), 10 mM HEPES pH 7.3, 1 mM sodium pyruvate, 1× non-essential amino acids, and 100 U/ml of penicillin–streptomycin. SARS-CoV-2/USA_WA1/2019 isolate expressing nanoluc luciferase (nLuc) was obtained from Craig B Wilen, Yale University and generously provided by K. Plante and Pei-Yong Shi, World Reference Center for Emerging Viruses and Arboviruses, University of Texas Medical Branch)^34^. Viruses were propagated in Vero E6 TMPRSS2 by infecting them in T150 cm^2^ flasks at a MOI of 0.1. The culture supernatants were collected after 18 h when cytopathic effects were clearly visible. The cell debris was removed by sedimentation and filtered through 0.45-micron filter to generate virus stocks. Viruses were concentrated by adding one volume of cold (4 °C) 4x PEG-it Virus Precipitation Solution [40 % (w/v) PEG-8000 and 1.2 M NaCl; System Biosciences] to three volumes of virus-containing supernatant. The solution was mixed by inverting the tubes several times and then incubated at 4°C overnight. The precipitated virus was harvested by centrifugation at 1,500 × g for 60 minutes at 4°C. The concentrated virus was then resuspended in PBS then aliquoted for storage at −80°C. All work with infectious SARS-CoV-2 was performed in Institutional Biosafety Committee of Yale University (IBCYU) approved BSL3 and A-BSL3 facilities at Yale University School of Medicine using appropriate positive pressure air respirators and protective equipment.

### ELISA

Ninety-six well Maxisorp plates (Nunc, Thermo Fisher Scientific) were coated overnight with 200 ng/well of purified recombinant RBD in PBS at 4°C. The day after, the plate was blocked with 100 ml/well of PBS+1% BSA for 30 minutes at room temperature. Mice sera were threefold serially diluted in PBS+1% BSA starting from a 1:100 initial dilution. After 3 washes with PBS+0.05% Tween 20 (PBST), 100 μl of each serum dilution were added in each well and the plate was incubated at room temperature for 1 hour. Wells were washed three times with PBST and then incubated 45 min at room temperature with goat anti mouse alkaline phosphatase-conjugate antibodies at a final dilution of 1:2000 (SigmaAldrich). After 3 washes with PBST, 100 ml/well of 3 mg/ml paranitrophenyl-phosphate disodium hexahydrate (Sigma Aldrich) in 1 M diethanolamine buffer pH 9.8 and plates were incubated at room temperature in the dark for 45min. Finally, absorbance was read at 405 nm using Varioskan^™^ LUX multimode microplate reader.

### Preparation of viral pseudotypes and neutralization assays

Lentiviral particles pseudotyped with SARS-Cov-2 spike were produced in 10-cm plates prepared the day before transfection with 3 million HEK293T cells in 10 ml complete DMEM, supplemented with 10% FBS. SIV-based vectors were produced by transfecting cells using the Calcium Phosphate method with 15 μg of env-defective SIV-Mac239-GFP construct with GFP expressed in place of Nef (PMID 26181333) and 1.5 μg of PCDNA3.1 encoding the WT SARS-CoV-2 spike (reference sequence Wuhan-Hu-1, accession number YP_009724390) engineered to truncate the C-terminal 19 amino acids. Pseudotyped vector supernatants were harvested 48 h post-transfection and filtered through a 0.45-μm filter before. Neutralization titres were tested on Huh-7 cells overexpressing ACE2. Target cells were seeded onto 96-well tissue culture one day before neutralization. The vectors inoculum was adjusted to produce no more than 10% transduction of the monolayer to ensure a linear working range of the assay.

Sera dilutions were added to pseudotyped virus particles, incubated at room temperature for 30 minutes and added to cells. After 48 h, transduction was assessed by counting the GFP-expressing cells using the fluorescent plate reader Ensight (Perkin Elmer). Each serum dilution was assessed in triplicate. Neutralization was measured by calculating the residual transduction activity of the pseudovirus considering the untreated sample as 100%. Fitted sigmoidal curves and IC50 were obtained using Prism (Graphpad) with the least square variable slope method and using the dose-normalized response protocol.

## Supplementary Material

1

## Figures and Tables

**Table 1:** 

Name	Nucleotide Sequence
1-F	TAATTAAAGCTGCAAAAGATATTAGCACCGAAATTTATCAGGC
1-R	GATGGTGATGGTGATGTTAACGATACGGCTGATAACCCAC
2-F	TAATTAAAGCTGCAAAAAGCAATAACCTGGATAGCAAAG
2-R	GATGGTGATGGTGATGTTATTTCAGATTGCTCTTACGAAAC
F-1	CCGTTTGAACGTGATATTAGCACCGAAATTTATCAGG
PACYC-F	AGCCAGGATCCGAATTCGAGC
FhuD2-v-R	TTTTGCAGCTTTAATTAATTTTTC
pET-v-F	CATCACCATCACCATCACGATTACA
R-1	ACGTTCAAACGGTTTCAGATTGCTCTTACGAAAC

**Table 2:** 

RBD_438-462_	AGCAATAACCTGGATAGCAAAGTTGGTGGCAACTATAACTATCTGTATCGCCTGTTTCGTAAGAGCAATCTGAAA
RBD_467-509_	GATATTAGCACCGAAATTTATCAGGCAGGTAGCACCCCGTGCAATGGTGTTGAAGGTTTTAATTGTTATTTTCCGCTGCAGAGCTATGGTTTTCAGCCGACAAATGGTGTGGGTTATCAGCCGTATCGT
RBD_438-509_	AGCAATAACCTGGATAGCAAAGTTGGTGGCAACTATAACTATCTGTATCGCCTGTTTCGTAAGAGCAATCTGAAACCGTTTGAACGTGATATTAGCACCGAAATTTATCAGGCAGGTAGCACCCCGTGCAATGGTGTTGAAGGTTTTAATTGTTATTTTCCGCTGCAGAGCTATGGTTTTCAGCCGACAAATGGTGTGGGTTATCAGCCGTATCGT
RBD-BA.1_438-509_	AGCAATAAACTGGATAGCAAAGTTAGCGGCAACTATAACTATCTGTATCGCCTGTTTCGTAAGAGCAATCTGAAACCGTTTGAACGTGATATTAGCACCGAAATTTATCAGGCAGGTAACAAACCGTGCAATGGTGTTGCGGGTTTTAATTGTTATTTTCCGCTGCGTAGCTATAGCTTTCGCCCGACATATGGTGTGGGTCATCAGCCGTATCGT

**Table 3:** 

Name	Clone	Source and catalog number
CD103	2E7	Biolegend #121407
MHCII I-a/I-b	M5/114.15.2	Biolegend #107622
Ly-6C	HK1.4	Biolegend #128026
CD11b	M1/70	Thermo Fisher #48-0112-82
SIGLEC F	E50-2440	BD Bioscences #740388
CD64	X54-5/7.1	BD OptiBuild #740622
CD8	53-6.7	Biolegend #100759
CD11c	HL3	BD Bioscences #563735
CD4	RM4-5	BD Bioscences #740208
CD44	IM7	BD Bioscences #741227
CD69	H1.2F3	BD Bioscences #612793
F4/80	BM8	Biolegend #123110
Ly6g	1A5	BD Pharmingen #562700
CD45	30-F11	Biolegend #103113

## Data Availability

All data supporting the findings of this study are available within the paper and its Supplementary Information.
