# Supplementary material for: Immunogenicity and pre-clinical efficacy of an OMV-based SARS-CoV-2 vaccine
Source: Res Sq. 2023 May 25:rs.3.rs-2788726. Preprint. [Version 1] doi: 10.21203/rs.3.rs-2788726/v1 (PMC10246226; doi:10.21203/rs.3.rs-2788726/v1)
Supplement: 1 [file NIHPPrs2788726v1-supplement-1.pdf]

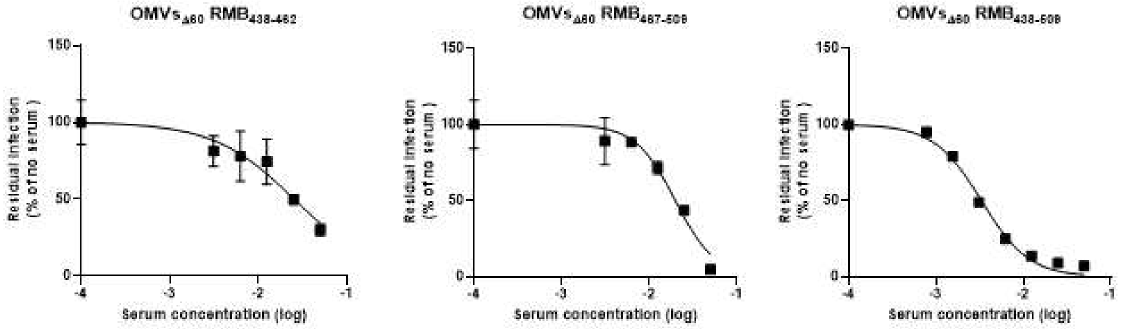

Figure S1. Neutralization activity against Ancestral SARS-CoV-2 isolate of sera from mice immunized with OMVs decorated with SARS-CoV-2 RBM$_{ancestral}$ antigens

Neutralization activity in pooled sera, measured with lentiviral vectors pseudotyped with SARS-CoV-2 spike from the ancestral isolate, plated on Huh-7 cells. Residual infectivity after treatment with serially diluted sera, expressed as percentage of the untreated virus control.

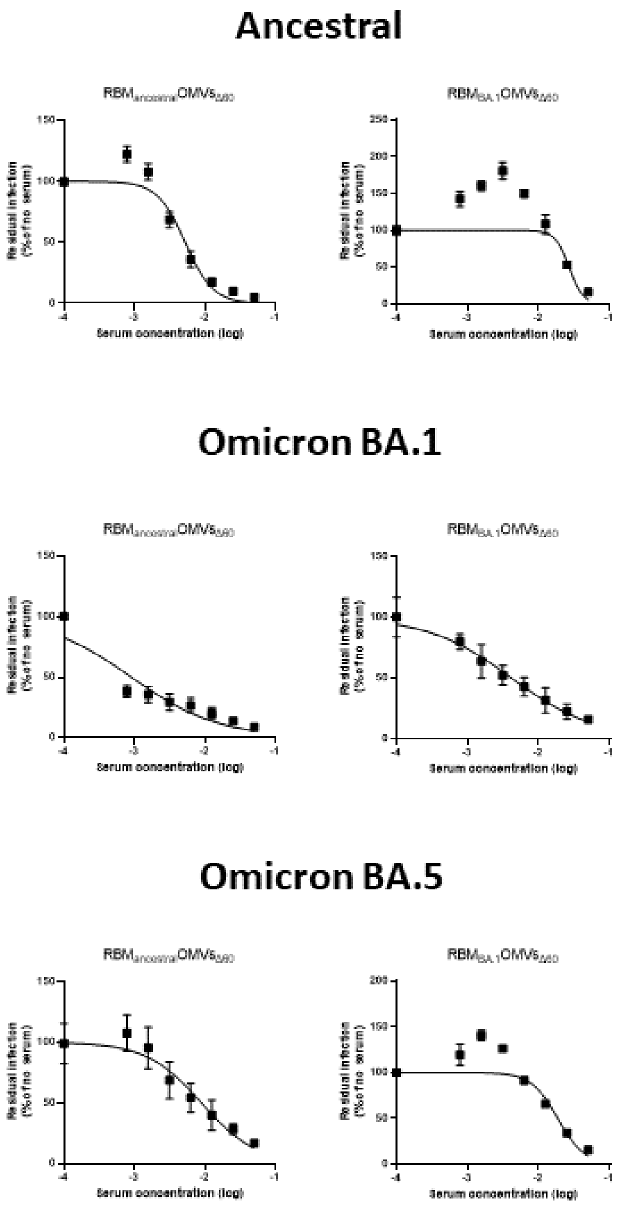

Figure S2. Cross-neutralizing activity of sera from mice immunized with OMVs decorated with SARS-CoV-2 RBM antigens

Neutralization activity in pooled sera derived from immunization with OMVs decorated with SARS-CoV-2 RBM antigens from the ancestral and omicron BA.1 strains, measured with lentiviral vectors pseudotyped with SARS-CoV-2 spike from the ancestral isolate and the omicron BA.1 and BA.5 isolates, plated on Huh-7 cells. Residual infectivity after treatment with serially diluted sera, expressed as percentage of the untreated virus control. Plotted are average values and standard deviations from triplicate determinations.

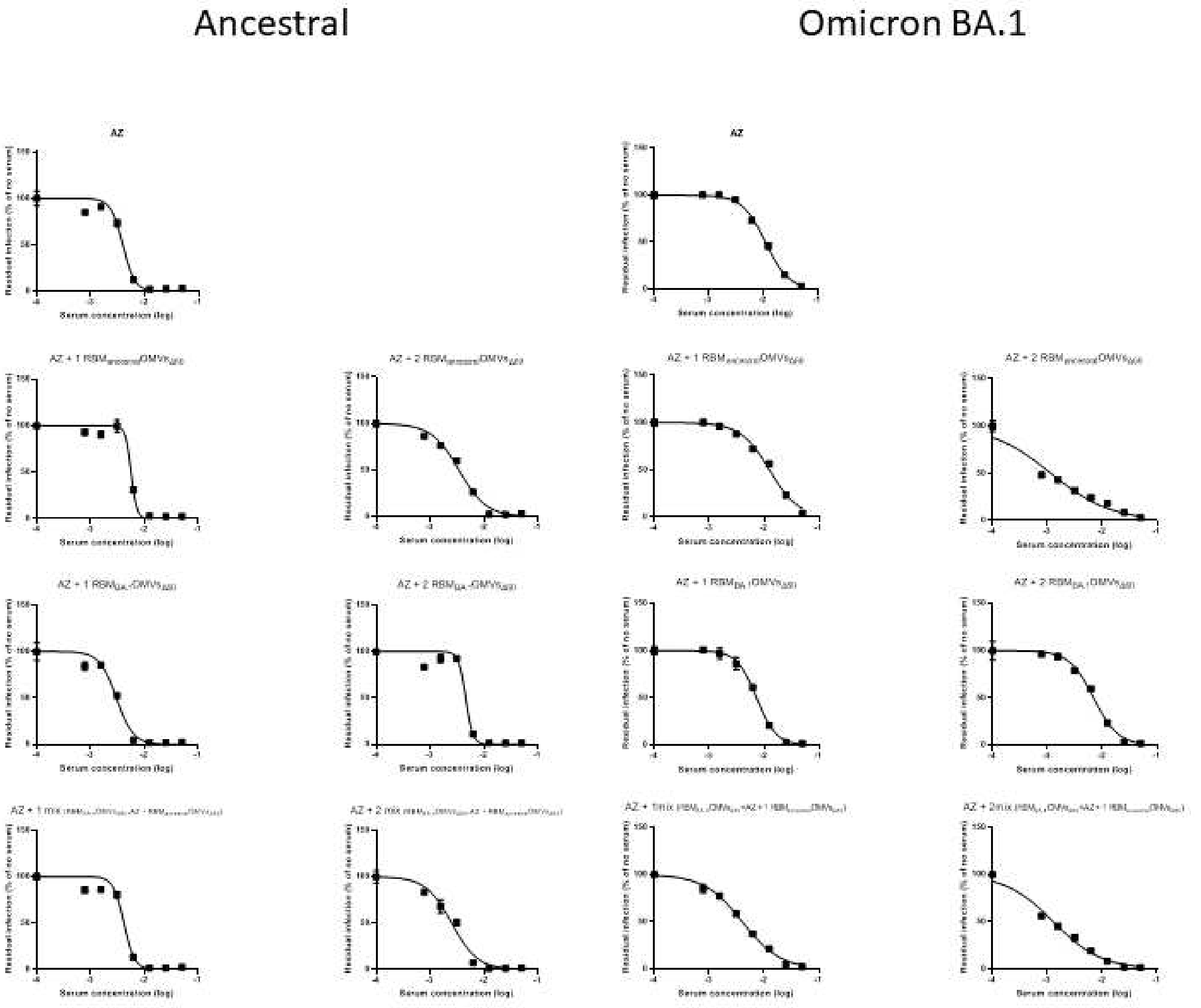

Figure S3. Ability of OMVs decorated with RBM antigens derived from the ancestral and omicron BA.1 isolates to boost immunity previously elicited by ChAdOx1(AZ)
Neutralization activity in sera from animals immunized following the experimental setup in figure 6B, measured with lentiviral vectors pseudotyped with SARS-CoV-2 spike from the ancestral isolate and the omicron BA.1 variant, plated on Huh-7 cells. Plotted are average values and standard deviations from triplicate determinations.
